# UbiNet 2.0: a verified, classified, annotated and updated database of E3 ubiquitin ligase–substrate interactions

**DOI:** 10.1093/database/baab010

**Published:** 2021-03-08

**Authors:** Zhongyan Li, Siyu Chen, Jhih-Hua Jhong, Yuxuan Pang, Kai-Yao Huang, Shangfu Li, Tzong-Yi Lee

**Affiliations:** School of Life and Health Sciences, The Chinese University of Hong Kong, Shenzhen, Guangdong 518172, P.R.China; School of Life and Health Sciences, The Chinese University of Hong Kong, Shenzhen, Guangdong 518172, P.R.China; Warshel Institute for Computational Biology, The Chinese University of Hong Kong, Shenzhen, Guangdong 518172, P.R.China; School of Science and Engineering, The Chinese University of Hong Kong, Shenzhen, Guangdong 518172, P.R.China; School of Life and Health Sciences, The Chinese University of Hong Kong, Shenzhen, Guangdong 518172, P.R.China; Warshel Institute for Computational Biology, The Chinese University of Hong Kong, Shenzhen, Guangdong 518172, P.R.China; Warshel Institute for Computational Biology, The Chinese University of Hong Kong, Shenzhen, Guangdong 518172, P.R.China; School of Life and Health Sciences, The Chinese University of Hong Kong, Shenzhen, Guangdong 518172, P.R.China; Warshel Institute for Computational Biology, The Chinese University of Hong Kong, Shenzhen, Guangdong 518172, P.R. China

## Abstract

Ubiquitination is an important post-translational modification, which controls protein turnover by labeling malfunctional and redundant proteins for proteasomal degradation, and also serves intriguing non-proteolytic regulatory functions. E3 ubiquitin ligases, whose substrate specificity determines the recognition of target proteins of ubiquitination, play crucial roles in ubiquitin–proteasome system. UbiNet 2.0 is an updated version of the database UbiNet. It contains 3332 experimentally verified E3–substrate interactions (ESIs) in 54 organisms and rich annotations useful for investigating the regulation of ubiquitination and the substrate specificity of E3 ligases. Based on the accumulated ESIs data, the recognition motifs in substrates for each E3 were also identified and a functional enrichment analysis was conducted on the collected substrates. To facilitate the research on ESIs with different categories of E3 ligases, UbiNet 2.0 performed strictly evidence-based classification of the E3 ligases in the database based on their mechanisms of ubiquitin transfer and substrate specificity. The platform also provides users with an interactive tool that can visualize the ubiquitination network of a group of self-defined proteins, displaying ESIs and protein–protein interactions in a graphical manner. The tool can facilitate the exploration of inner regulatory relationships mediated by ubiquitination among proteins of interest. In summary, UbiNet 2.0 is a user-friendly web-based platform that provides comprehensive as well as updated information about experimentally validated ESIs and a visualized tool for the construction of ubiquitination regulatory networks available at http://awi.cuhk.edu.cn/~ubinet/index.php.

## Introduction

The ubiquitin–proteasome system (UPS) controls protein turnover by labeling malfunctional and redundant proteins for proteasomal degradation. This particular process, carried out by a cascade consisting of ubiquitin activating enzyme (E1), ubiquitin conjugating enzyme (E2) and ubiquitin ligase (E3), is called ubiquitination. Ubiquitination is involved in significant cellular activities such as the regulation of protein expression, activity of receptors and endocytosis, and antigen presentation. In the last two decades, it has drawn growing attention because of its significant role in a plethora of diseases, including cancers, neurodegenerative diseases, autoimmune diseases and many others (1, 2). In ubiquitination process, E3 ubiquitin ligases are the crucial enzymes that are in charge of the recognition of ubiquitination substrates. According to this feature, some degradants have been applied to hijack the cell degradation mechanism by recruiting E3 ubiquitin ligase to the proteins of interest, forming targeted protein degradation (TPD), which has been considered as an emerging therapeutic modality (3). Therefore, research on the specificity of E3 and substrates may provide inspiration for drug discovery and new therapies (4, 5).

The biological significance of E3 ligase in cellular processes has driven research in related fields of bioinformatics. Some databases and tools have been developed to study the E3 ligases. For example, E3Net provided a comprehensive resource of E3 ligases, substrates and their functional implications (6). Chen *et al.* built a computational model for predicting E3 ligase and substrate interactions, offering an omics-driven way to portray the E3–substrate association patterns (7). UbiBrowser developed an integrated bioinformatics platform to predict and present the proteome-wide human E3–substrate interaction (ESI) network (8). Our group also constructed a database, UbiNet 1.0, to explore the functional associations and regulatory networks of protein ubiquitylation (9). The database contained 43 948 ubiquitination sites from 14 692 human ubiquitylated proteins and 499 non-redundant E3 ubiquitin ligases. In addition, UbiNet 1.0 also provides an interactive network viewer as well as the functional and structured annotations of these ubiquitylation-related proteins, such as physical and chemical properties, diseases and pathways involved, protein–protein interactions (PPIs) and substrate motifs at ubiquitination sites. Currently available ubiquitination-related resources promote the understanding of the function and regulation of ubiquitination modification. However, the vigorous development of studies on ubiquitination has resulted in the continuous generation of new E3–substrate data, which requires timely collection and sorting. In addition, the simple aggregation of these data is not friendly to the systematic research and use of these ubiquitination data. Therefore, it is necessary to carry out a reasonable classification based on the structure and functional characteristics of E3 ligase to facilitate the utility of ubiquitination modification data and portray a comprehensive E3–substrate landscape.

Therefore, we have now updated and improved UbiNet to make it a comprehensive database of verified, classified and visualized ESIs. We collected experimentally verified relationships between substrates and specific E3 ligases from various sources. Each E3 and substrate was annotated thoroughly, and the recognition motifs in substrates for each E3 were also investigated. In addition, the E3 ligases in the database were classified strictly based on the mechanism of ubiquitin transfer and substrate specificity. These updates have been integrated into UbiNet 2.0, forming a unique resource and providing an extensive and informative knowledge base for ubiquitination. UbiNet 2.0 will be very valuable for exploring the interaction of E3–substrates and can also meet the needs of a wider range of research interests.

### The system flow of UbiNet 2.0

**Figure 1.**
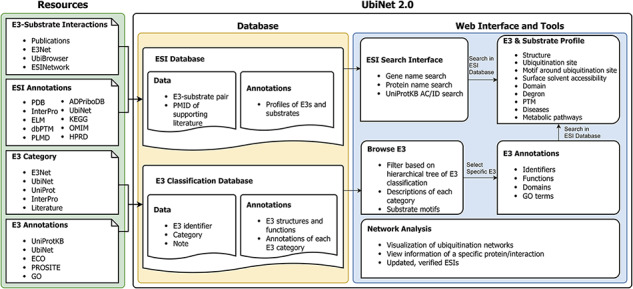
Schematic representation of the data integration and database construction and the development of web interface and analytics tools of UbiNet 2.0.

Based on the previous version, we have made a major update to UbiNet. Figure 1 shows the overall structure of UbiNet 2.0. We collected ESI data and their annotations from public databases and literature publications, forming the ESI database. In addition, we also classified and annotated the E3s and saved the results in the E3 classification database. Both databases can be accessed and retrieved through UbiNet 2.0’s web interface and tools (Figure 1).

### ESI data collection, processing and organization

**Figure 2.**
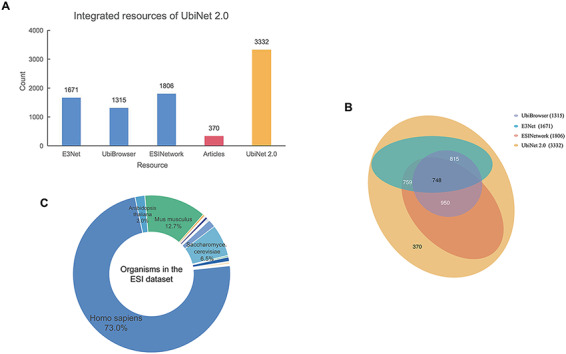
Integrated resources of UbiNet 2.0. (A) The numbers of ESIs collected from three other platforms, literature as well as UbiNet 2.0. (B) The Venn diagram on the resources. It shows the overlap of ESI records between the resources. The numbers in the brackets beside the database names are the total number of ESIs in the corresponding database. (C) Organisms involved in the ESI data set of UbiNet 2.0.

All the experimentally verified ESIs and the relationships between E3s and their specific substrates in different organisms were collected from various sources. (Figure 2A). The ESIs from other platforms, including E3Net (6), UbiBrowser (8) and ESINetwork (7), were fully integrated into UbiNet 2.0. The Venn diagram (Figure 2B) showed the overlap between UbiNet 2.0 and these resources. In addition, newly discovered ESIs were curated from 1241 articles published between January 2019 and 8 June 2020 by searching against PubMed. The keywords were based on Li’s work (8) listed inSupplementary Table S1. Then, the retrieved articles were manually reviewed to extract the experimentally validated ESIs, organisms and PubMed ID. A total of 370 ESIs were extracted from the articles and integrated with ESIs from other platforms to construct a non-redundancy ESI data set. The de-redundancy was based on the most recent version of UniProtKB accession number (10). Finally, 3332 unique ESIs were included in the data set. Compared to UbiNet 1.0, which collected the ubiquitination information only from studies on human samples, UbiNet 2.0 expanded the list up to 54 organisms. Among them, human origin accounted for the majority, reaching 73%. Rats, mice, yeast and other organisms were followed (Figure 2C). This greatly improved the integrity and availability of the database, considering that more and more experiments were conducted on model organisms. The data statistics on the numbers of substrates for each organism and for each E3, as well as the numbers of E3s and ESIs for each organism, are provided in Supplementary Tables S2, S3 and S4.

### E3 and substrate profiles

The profiles of the E3 and the substrate were provided for each ESI with the fundamental structural and functional annotations. The information included 3D protein structures from the Protein Data Bank (11), protein domains from InterPro (12), PPI and ubiquitination sites summarized by UbiNet 1.0, metabolic pathways from Kyoto Encyclopedia of Genes and Genomes (KEGG) (13), and related diseases incorporated from KEGG, Online Mendelian Inheritance in Man (14) and Human Protein Reference Database (15).

In addition, UbiNet 2.0 added annotations that are related to the regulation of ubiquitination network and the substrate specificity of E3s. The annotations included the degrons in the form of a short linear sequence from the eukaryotic linear motif resource (16) and phosphorylation, neddylation (17) and PARylation (18) from dbPTM (19), PLMD (1, 20) and ADPriboDB (21), respectively. All the above information has been embodied in new profiles (Supplementary Materials-1 Table S1).

### E3 classification

**Figure 3.**
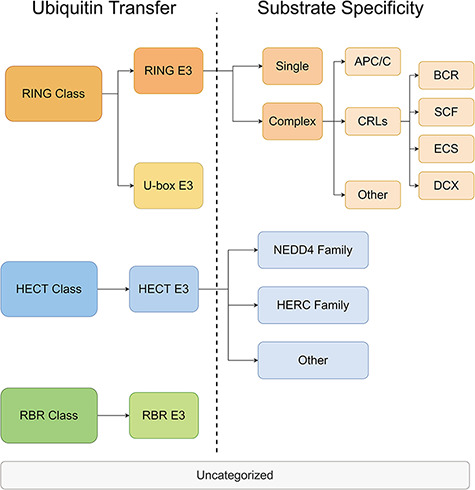
The hierarchical tree of E3 categories.

All E3s in the ESI database were classified and the information was stored in a separate database. The classification was performed on two levels. On the first level, the E3 ligases were divided into three major classes based on the mechanisms of ubiquitin transfer, namely RING, HECT and RBR (22). Then, according to the characteristic domains, the three major classes were further divided into four minor classes, namely RING E3s, U-box E3s, HECT E3s and RBR E3s. On the second level, E3s were categorized with distinct mechanisms of substrate recognition. The rules for classification were customized for each minor class from the first level. RING E3s contains two forms—single polypeptide and E3 complex (23). The RING E3 complexes have a large number of members, including anaphase-promoting complex (APC/C), cullin-RING ligases (CRLs) and other complexes. Among them, CRLs are the largest family of E3 with different substrate-recognition modules discovered for complexes with different cullin domains (24). The HECT E3s were classified into NEDD4 family, HERC family and other HECT E3s, according to their conformationally diverse N-terminal (25). U-box E3s and RBR E3s were not classified on the second level because there were only a few members within the classes. The categories and the annotations of E3s are listed in Figure 3 and Supplementary Materials-1 Table S1.

### Recognition motifs in substrates

The specific recognition between E3 ligase and substrates is a key step in ubiquitination modification (22). Explaining the specificity is an important topic in the study of ubiquitination. Analysis of substrate motifs is an important way to study the specific recognition between E3 ligase and substrate. Specifically, owing to the high throughput of mass spectrometry–based proteomics, a large number of ESIs have been discovered, which provide abundant materials for the exploration of the recognition motifs for human E3s. GLAM2 (26) was applied to discover potential motifs that might be recognized by a specific E3 ligase among its substrates. Before that, to obtain an unbiased motif analysis, the homologous sequences were removed from the substrates for each E3 by using Cluster Database at High Identity with Tolerance program (27). Based on a previous relevant report (28), the motif length was limited within 3–10 residues.

Herein, we analyzed the specific substrate motifs of 109 human E3s that had at least five substrates. Some of the substrate motifs have been reported, which were consistent with previous results. For instance, we identified KEN motif presented in 52 substrates of E3 ligase CDH1 (Supplementary Materials-1 Figure S1A). It has been reported that KEN box serves as a general targeting signal for Cdh1 (29). The substrates identified through KEN motif included SCL-interrupting locus protein STIL that played an important role in the regulation of centriole duplication (30), histone acetyltransferase component TRRAP (31), and so on. In addition, we investigated the substrates of FBXW7, which is the substrate recognition component of an SCF (SKP1–CUL1–F-box protein)–E3 ubiquitin-protein ligase complex, and found a TPPxS motif existing in 48 substrates of FBXW7 (Supplementary Materials-1 Figure S1B). The result was validated by a review paper, which demonstrated that FBXW7 recognized the phosphodegron pTPPxS motif (where p indicates a phosphorylated residue) in substrates important for cell growth (32).

Another example is Nedd4 family of E3 ubiquitin ligases such as NEDD4, NEDD4-2, ITCH, SMURF1, WWP1, WWP2 and SMURF2. As evidenced by previous reports, Nedd4 family proteins were found throughout eukaryotes and regulate diverse biological processes through the targeted degradation of proteins that generally have a PPxY motif for WW domain recognition (33). We actually identified PPxY motif that was more likely to occur in proline-rich regions as the most significant motifs for some members of Nedd4 family such as WWP1 (Supplementary Materials-1 Figure S1C). There were also non-PPxY recognition motifs for some members of Nedd4 family such as in the case of SMURF2 (motif RLxxELE, Supplementary Materials-1 Figure S1D), which is in line with the previous results that the presence of PPxY motifs is not necessary for Nedd4 family E3s to recognize the proteins. They can target the substrates containing PPxY motifs as well as those without the motifs or with other unknown motifs (34, 35). All the recognition motifs were identified and the related information for each E3 was provided in UbiNet 2.0.

### Functional enrichment analysis on substrates

**Figure 4.**
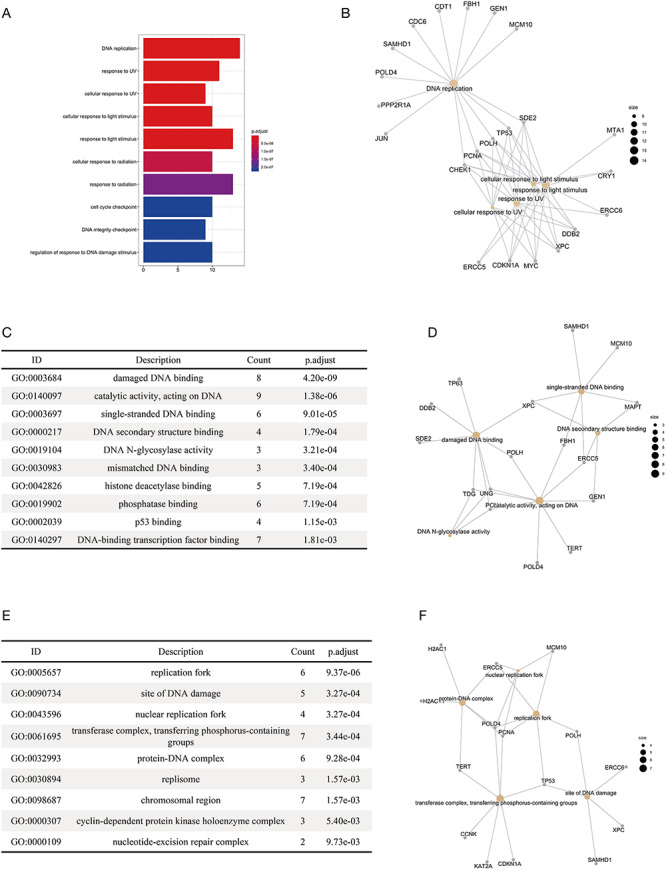
The results of the functional enrichment analyses on the substrates of DCX E3 ligases. (A) The bar chart shows the enriched biological processes of the substrates of DCX E3s. (B) The network between the substrates of DCX E3s and the enriched biological processes. The yellow circles represent the functional terms, while the gray circles represent the substrates. The size of the yellow circles indicates the number of substrates associated with that functional term. (C) The bar chart illustrates the enriched molecular functions of the substrates of DCX E3s. (D) The network between the substrates of DCX E3s and the enriched molecular functions. (E) The enriched cellular compartments of the substrates of DCX E3s are listed. (F) The network between the substrates of DCX E3s and enriched cellular compartments.

We performed functional enrichment analysis on the substrates of E3 ligases in different categories, including APC/C, BCR, DCX, ECS, UBOX, NEDD4, HERC, SCF and RBR. The gene ontology analysis (36, 37) and disease analysis (38) were conducted using an R package ‘clusterProfiler’ (39), with the *P*-values adjusted by the Benjamini–Hochberg Procedure. The enrichment of KEGG pathways was conducted using another R package ‘gprofiler2’, with the g:SCS algorithm being the correction method (40). Distinct results were obtained for substrates of different E3 categories. For example, we found that the enriched biological processes of the substrates were mostly related to the response to UV lights and DNA replication (Figure 4A). The result was highly consistent with the previous reports that the substrates of DCX (DDB–CUL4–X box)–E3s were significantly associated with UV-light-induced DNA damage, nucleotide excision repair (NER) (41) and DNA replication (42). Figure 4B clearly demonstrated the substrates that were specific for each function (e.g. CDC6 and JUN are specific for DNA replication) and those common to different functions (e.g. CHEK1, PCNA, POLH and TP53). The enrichment of molecular functions revealed the association of the substrates of DCX E3s with DNA binding and binding to proteins involved in NER such as DNA N-glycosylase (Figure 4C and D). The enriched cellular compartments included the site of DNA replication and DNA damage (Figure 4E and F), which was consistent with the enriched biological processes and molecular functions. We also made interesting discoveries on the substrates of BCR (BTB box–CUL3–RBX1)–E3s, which, according to our results, were highly correlated with the development of cancers of reproductive system (Supplementary Materials-2). All the results of functional enrichment analysis could be found in Supplementary Materials-2.

### Comparison with other platforms

Due to the biological significance of ubiquitination, the experimental studies on ESIs have been continuously carried out. This promotes the development of some databases or forecasting tools, such as E3Net, ESINetwork, UbiBrowser, etc. Compared with these platforms, UbiNet 2.0 was unique in the following aspects (Table 1). First, the verified ESIs included in UbiNet 2.0 were currently the latest and most comprehensive ESIs, reaching 3332 records. Among other platforms, ESINetwork had the most ESIs with 1806 records but only 54% of UbiNet 2.0. Secondly, these ESIs of UbiNet 2.0 were collected from 54 organisms, 22% more than E3Net, which was the largest among other platforms. It meant that the organisms included in our database were currently the most comprehensive ones. In addition, the classification of E3 was also what made our database different from other platforms. E3Net was the most detailed database with classified information among other platforms. It was classified based on subunit composition and domain subclass. In UbiNet 2.0, E3s are classified according to distinct mechanisms of ubiquitin transfer and substrate recognition. This made the classification more specific and reasonable. Being informative and constructive, UbiNet 2.0 provided descriptions of each category as well as functional and structural annotations of every classified E3. The reasonable classification and annotations may facilitate the discovery of common interaction and catalytic properties of a specific E3 class.

**Table 1.** Comparison between UbiNet 2.0 and other platforms

Feature	UbiNet 2.0	E3Net	UbiNet 1.0	ESINetwork	UbiBrowser
Number of ESIs	3332	1671	None	1806	1315
Organisms	54	42	1	1	1
Latest date of data updating	Before 8 June 2020	Before April 2012	Before March 2016	Before June 2019	Before August 2017
Database content	Verified E3–substrate pairs	Verified E3–substrate pairs	PPIs	Verified E3–substrate pairs	Verified E3–substrate pairs
Principle of E3 classification	Mechanisms of ubiquitin transfer and substrate specificity	Subunit composition and domain subclass	RING, HECT or other	Unavailable	Unavailable
Annotations of E3 Categories	Available	Unavailable	Unavailable	Unavailable	Unavailable

### Web interface and utility

**Figure 5.**
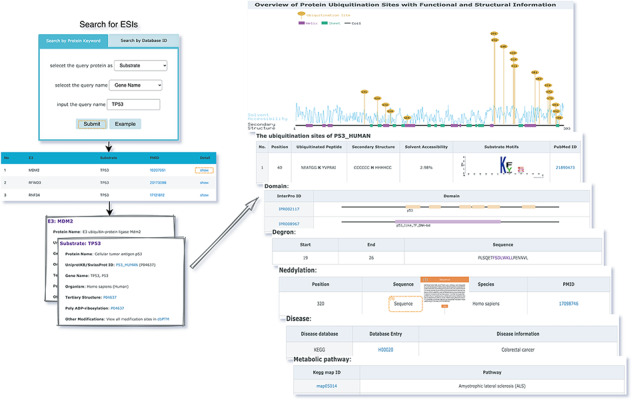
The first service for browsing ESIs by specifying identifiers of an E3 or a substrate in the search panel and view the E3 and substrate profiles.

**Figure 6.**
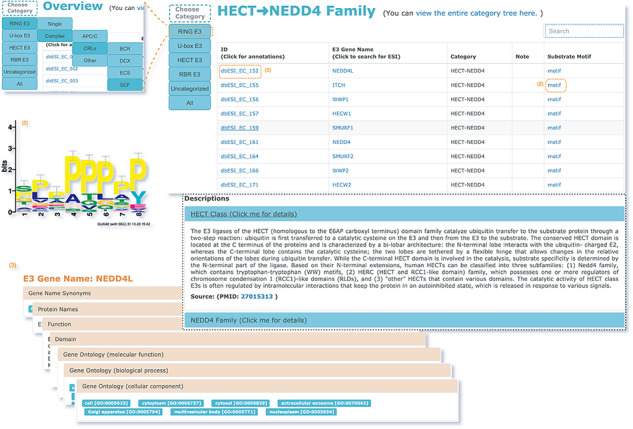
The second service for browsing the data of E3 classification.

**Figure 7.**
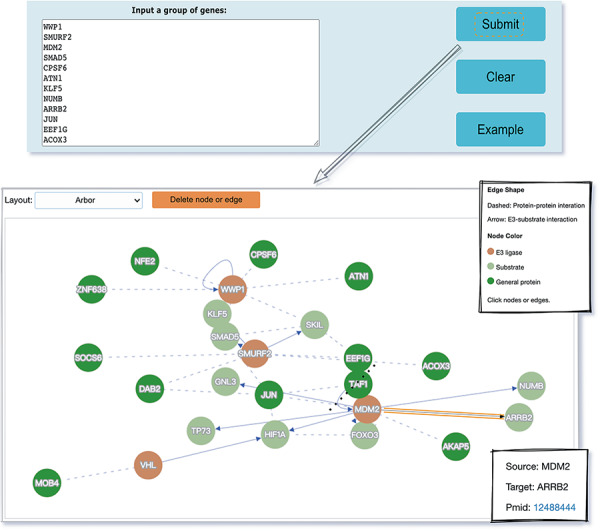
The visualization tool used to generate a network of a list of proteins specified by users.

The web interface is composed of three major services. The first service allows users to browse ESIs by specifying identifiers of an E3 or a substrate in the search panel, to obtain a list of related experimentally validated ESIs with supporting literatures and to view the E3 and substrate profiles hyperlinked to each ESI (Figure 5). The ‘Organism’ option in the search result page is provided to allow users to browse the ESIs of specific substrate or E3 in the selected organism. The second service is designed for browsing the data of E3 classification. A filter can guide users to a page of a certain category containing general descriptions and a table summarizing the E3s, inside which the hyperlinked ID of an E3 guides users to a page presenting the evidence used for classification and additional annotations. The substrate motifs for E3s can be accessed by clicking on the ‘Motif’ button in the table. Users can also search for ESIs of a particular E3 via simply clicking on the gene name of that E3 (Figure 6). The third service is a visualization tool that generates a network of a list of proteins specified by users based on the physical PPIs and verified E3–substrate relationships among the given proteins. For the physical PPI data set, seven related databases were integrated, including Mentha, IntAct, InteroPorc, BIOGRID, APID, MINT and Reactome (Supplementary Table S1). If there are verified E3–substrate relationships or physical PPIs among provided proteins, there will be edges linking them. Within the network, a protein is represented by a node and an interaction is represented by an edge. By clicking on a node or an edge, users can view the detailed information of the protein or the interaction. If the proteins specified by users are all E3s, the tool will automatically display networks consisting of these E3s and all the corresponding substrates (Figure 7). This tool allows researchers to analyze the ubiquitination network among a large group of proteins of interest without first obtaining knowledge about each individual protein, which indicates the potential regulatory relationship between these proteins.

## Conclusion and future perspective

Since the publication of original UbiNet, an increasing number of experimentally validated ESIs have been reported. The cumulative data allowed us to construct an updated UbiNet (UbiNet 2.0), where ESIs could be supported by experimental evidence from extrapolation of the physical interaction between E3s and ubiquitinated proteins. This greatly improved the reliability and availability of the platform. Based on these data, we classified E3s and their corresponding substrates more reasonably, so as to look into the specific relationships between E3s and substrates. In UbiNet 2.0, each E3 and substrate was annotated thoroughly and the recognition motifs in substrates for each E3 were also investigated. Moreover, the platform can provide an interactive regulatory network mediated by ubiquitination and protein interaction for a group of proteins of interest to users. Abnormal ubiquitination is closely related to carcinogenesis. The planned future update will incorporate information on the contribution of ESIs to different types of cancer, which is significant for the researches on the mechanisms of cancers.

## Supplementary Material

baab010_Supp
