# Supplementary material for: UbiNet 2.0: a verified, classified, annotated and updated database of E3 ubiquitin ligase–substrate interactions
Source: Database (Oxford). 2021 Mar 8;2021:baab010. doi: 10.1093/database/baab010 (PMC7947570; doi:10.1093/database/baab010)
Supplement: baab010_Supp [file baab010_supp.zip › Supplementary Materials-1.doc]

Figure S1. The recognition motifs for CDH1, FBXW7, WWP1, and SMURF2. The square brackets in regular expressions mean: any one of the letters in the brackets. A dot means: any one letter.

**Table S1. Integrated annotation resources of UbiNet2.0.**

| Service | Resource | Data |
| --- | --- | --- |
| E3-substrate profiles | dbPTM | Common post-translational modifications |
|  | ADPriboDB | PARylation |
|  | ELM | Degrons |
|  | PLMD | Neddylation |
|  | UbiNet | Ubiquitination sites |
|  |  | Surface solvent accessibility of ubiquitylated peptides |
|  |  | Secondary structure of ubiquitylated peptides |
|  |  | Substrate motifs of ubiquitylation sites |
|  | InterPro | Domains |
|  | KEGG, OMIM, HPRD | Related diseases |
|  | KEGG | Metabolic pathways |
| E3 annotations | UniProtKB | Function |
|  | UbiNet, ECO, PROSITE | Domains |
|  | GO | Gene ontology terms |
|  | UniProtKB | Protein sequence |
