# Supplementary material for: UbiNet 2.0: a verified, classified, annotated and updated database of E3 ubiquitin ligase–substrate interactions
Source: Database (Oxford). 2021 Mar 8;2021:baab010. doi: 10.1093/database/baab010 (PMC7947570; doi:10.1093/database/baab010)
Supplement: baab010_Supp [file baab010_supp.zip › Supplementary Materials-2.doc]

Figure S2. The results of the functional enrichment analysis on the substrates of APC/C E3 ligases. A) The bar chart shows the enriched biological processes of the substrates of APC/C E3s. B) The network between the substrates of APC/C E3s and the enriched biological processes. The yellow circles represent the functional terms, while the grey circles represent the substrates. The size of the yellow circles indicates the number of substrates associated with that functional term. C) The bar chart illustrates the enriched molecular functions of the substrates of APC/C E3s. D) The network between the substrates of APC/C E3s and the enriched molecular functions. E) The enriched cellular compartments of the substrates of APC/C E3s are listed. F) The network between the substrates of APC/C E3s and enriched cellular compartments. G) and H) The diseases to which the substrates of APC/C E3s are enriched, and the network between them.

Figure S3. The results of the functional enrichment analyses on the substrates of BCR E3 ligases. A) The bar chart shows the enriched biological processes of the substrates of BCR E3s. B) The network between the substrates of BCR E3s and the enriched biological processes. The yellow circles represent the functional terms, while the grey circles represent the substrates. The size of the yellow circles indicates the number of substrates associated with that functional term. C) The bar chart illustrates the enriched molecular functions of the substrates of BCR E3s. D) The network between the substrates of BCR E3s and the enriched molecular functions. E) The enriched cellular compartments of the substrates of BCR E3s are listed. F) The network between the substrates of BCR E3s and enriched cellular compartments. G) and H) The diseases to which the substrates of BCR E3s are enriched, and the network between them.

Figure S4. The results of the functional enrichment analyses on the substrates of DCX E3 ligases. A) The bar chart shows the enriched biological processes of the substrates of DCX E3s. B) The network between the substrates of DCX E3s and the enriched biological processes. The yellow circles represent the functional terms, while the grey circles represent the substrates. The size of the yellow circles indicates the number of substrates associated with that functional term. C) The bar chart illustrates the enriched molecular functions of the substrates of DCX E3s. D) The network between the substrates of DCX E3s and the enriched molecular functions. E) The enriched cellular compartments of the substrates of DCX E3s are listed. F) The network between the substrates of DCX E3s and enriched cellular compartments. G) and H) The diseases to which the substrates of DCX E3s are enriched, and the network between them.

Figure S5. The results of the functional enrichment analyses on the substrates of ECS E3 ligases. A) The bar chart shows the enriched biological processes of the substrates of ECS E3s. B) The network between the substrates of ECS E3s and the enriched biological processes. The yellow circles represent the functional terms, while the grey circles represent the substrates. The size of the yellow circles indicates the number of substrates associated with that functional term. C) The bar chart illustrates the enriched molecular functions of the substrates of ECS E3s. D) The network between the substrates of ECS E3s and the enriched molecular functions.

Figure S6. The results of the functional enrichment analyses on the substrates of HERC E3 ligases. A) and B) The diseases to which the substrates of HERC E3s are enriched, and the network between them.

Figure S7. The results of the functional enrichment analyses on the substrates of NEDD4 E3 ligases. A) The bar chart shows the enriched biological processes of the substrates of NEDD4 E3s. B) The network between the substrates of NEDD4 E3s and the enriched biological processes. The yellow circles represent the functional terms, while the grey circles represent the substrates. The size of the yellow circles indicates the number of substrates associated with that functional term. C) The bar chart illustrates the enriched molecular functions of the substrates of NEDD4 E3s. D) The network between the substrates of NEDD4 E3s and the enriched molecular functions. E) The enriched cellular compartments of the substrates of NEDD4 E3s are listed. F) The network between the substrates of NEDD4 E3s and enriched cellular compartments. G) and H) The diseases to which the substrates of NEDD4 E3s are enriched, and the network between them.

Figure S8. The results of the functional enrichment analyses on the substrates of RBR E3 ligases. A) The enriched cellular compartments of the substrates of RBR E3s are listed. B) The network between the substrates of RBR E3s and enriched cellular compartments. C) and D) The diseases to which the substrates of RBR E3s are enriched, and the network between them.

Figure S9. The results of the functional enrichment analyses on the substrates of SCF E3 ligases. A) The bar chart shows the enriched biological processes of the substrates of SCF E3s. B) The network between the substrates of SCF E3s and the enriched biological processes. The yellow circles represent the functional terms, while the grey circles represent the substrates. The size of the yellow circles indicates the number of substrates associated with that functional term. C) The bar chart illustrates the enriched molecular functions of the substrates of SCF E3s. D) The network between the substrates of SCF E3s and the enriched molecular functions. E) The enriched cellular compartments of the substrates of SCF E3s are listed. F) The network between the substrates of SCF E3s and enriched cellular compartments. G) and H) The diseases to which the substrates of SCF E3s are enriched, and the network between them.

Figure S10. The results of the functional enrichment analyses on the substrates of UBOX E3 ligases. A) The enriched cellular compartments of the substrates of UBOX E3s are listed. B) The network between the substrates of UBOX E3s and enriched cellular compartments. C) and D) The diseases to which the substrates of UBOX E3s are enriched, and the network between them.
